# Feasibility of Fish Oil Supplementation on Headache Symptoms and Blood Lipids in Migraine Patients

**DOI:** 10.1002/brb3.70149

**Published:** 2024-12-06

**Authors:** En‐Ling Yeh, Chun‐Pai Yang, Shu‐Wen Lin, Hsueh‐Fang Wang

**Affiliations:** ^1^ Department of Nutrition Hungkuang University Taichung Taiwan; ^2^ Department of Neurology Kuang Tien General Hospital Taichung Taiwan; ^3^ Department of Nutrition Kuang Tien General Hospital Taichung Taiwan

**Keywords:** blood lipid, fish oil, headache symptom, migraine

## Abstract

**Objectives:**

Migraine is a chronic, recurring, and disabling disease. Fish oil intervention was used to investigate its effects on headache symptoms and blood lipids of migraine patients.

**Design:**

All subjects were collected at the Kuang Tian General Hospital from March 2020 to May 2021. Experimental group subjects took 1 g/time of fish oil (including EPA 900 mg/tablet) after breakfast and dinner. Placebo group subjects took 100% soybean oil twice daily. Before and after the test, the migraine improvement questionnaire was used to analyze headaches during attacks, dietary intake, and headache triggers.

**Results:**

The average age of the 47 subjects in this study was 40.3 ± 9.2 years old, the body mass index (BMI) was 24.3 ± 6.0 kg/m^2^. At Week 12, subjects in the fish oil group were significantly improved relative to the control group (*p* < 0.05). *Blood lipid indexes TC, LDL‐C, and TG were reduced, and the frequency, duration, and pain degree of migraine*.

**Conclusion:**

Fish oil may be used as an adjunctive therapeutic food for relieving migraine attack symptoms and blood lipids.

## Graphical abstract

Blood lipid indexes TC, LDL‐C, and TG, were reduced, along with decreases in the frequency, duration, and intensity of migraine pain.

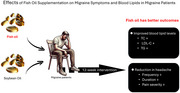

## 1 Introduction

Migraine is a recurring and disabling neurological disorder with symptoms such as nausea, vomiting, photophobia, or noise (Sprenger, Viana, and Tassorelli 2018; Yeh, Blizzard, and Taylor 2018). Migraine attacks not only affect patients themselves but also affect their family members and friends, and are more likely to cause social disruption, anxiety, and fear (Steiner et al. 2014). The world average prevalence of migraine is 14.4% (Burch et al. 2015; Razeghi Jahromi et al. 2019) and is the second most common disease in the world after dental caries and tension‐type headache (Steiner et al. 2020). It usually begins during adolescence and most commonly affects not only people aged 35–55 years but also some older adults (Bigal, Liberman, and Lipton 2006).

In Taiwan, the prevalence of migraine in females is two to three times higher than in males (Razeghi Jahromi et al. 2019) and the prevalence of migraine is 9.1% (14.4% in females and 4.5% in males). Severe migraine pains not only cause extreme discomfort to the patient but also affect their lives and are also a burden to the medical system. Common side effects of clinical migraine treatment drugs, such as drowsiness, palpitations, chest tightness, nausea, and vomiting, cause additional burden (Huang and Lai 2017). Studies have found that migraine is genetically related, with a 40% chance of having migraine in children if one parent has migraine and a 75% chance of having migraine if both parents have it (Peters 2019).

From a clinical viewpoint, precipitating factors seriously affect some migraine patients. Senses are sharpened during or before a migraine attack, such as smell for cigarettes, perfume, and bleach. Imai's study showed that odors are associated with migraine attacks and suggested that some chemicals are more likely associated with migraine attacks in patients (Imai et al. 2023). In addition, caffeinated foods, processed foods, drug abuse, changes in sleep habits, weather changes, changes in female hormones (ego, menstrual periods, menopause), obesity, stress, and neck pain can also trigger migraine headaches (Bigal, Liberman, and Lipton 2006; Imai et al. 2023).

Both clinical studies and animal experiments have demonstrated that omega‐3 polyunsaturated fatty acids (PUFAs) have anti‐inflammatory activity and beneficial effects in a variety of human inflammatory diseases, including diabetes, atherosclerosis, asthma, and arthritis (Munakata et al. 2009). Eicosapentaenoic acid (EPA) and docosahexaenoic acid (DHA) are omega‐3 fatty acids found in oily fish and fish oil supplements. The anti‐inflammatory properties of EPA are higher than those of DHA; EPA has been shown to reduce plasma total cholesterol (TC) and triglyceride (TG) values, and to help regulate blood pressure, blood lipids, and body weight (Cardia et al. 2020). It can effectively inhibit blood coagulation to prevent thrombosis and improve lipoprotein metabolism in patients with hyperlipidemia, so it can reduce blood cholesterol values (Goadsby et al. 2017; Imai et al. 2023). In addition, clinical trials have demonstrated the ability to modulate multiple pain‐related biochemical pathways, and dietary supplementation with omega‐3 PUFAs is beneficial in inflammatory and autoimmune diseases in humans (Weitz et al. 2010; Calder 2010), but still it is not sure in migraine improvement. Therefore, this study intends to intervene in migraine patients in a double‐blind manner and compares the effect on migraine in adults before and after intervention by using a structured questionnaire and blood biochemical values.

## 2 Methods

### 2.1 Participants

**FIGURE 1:**
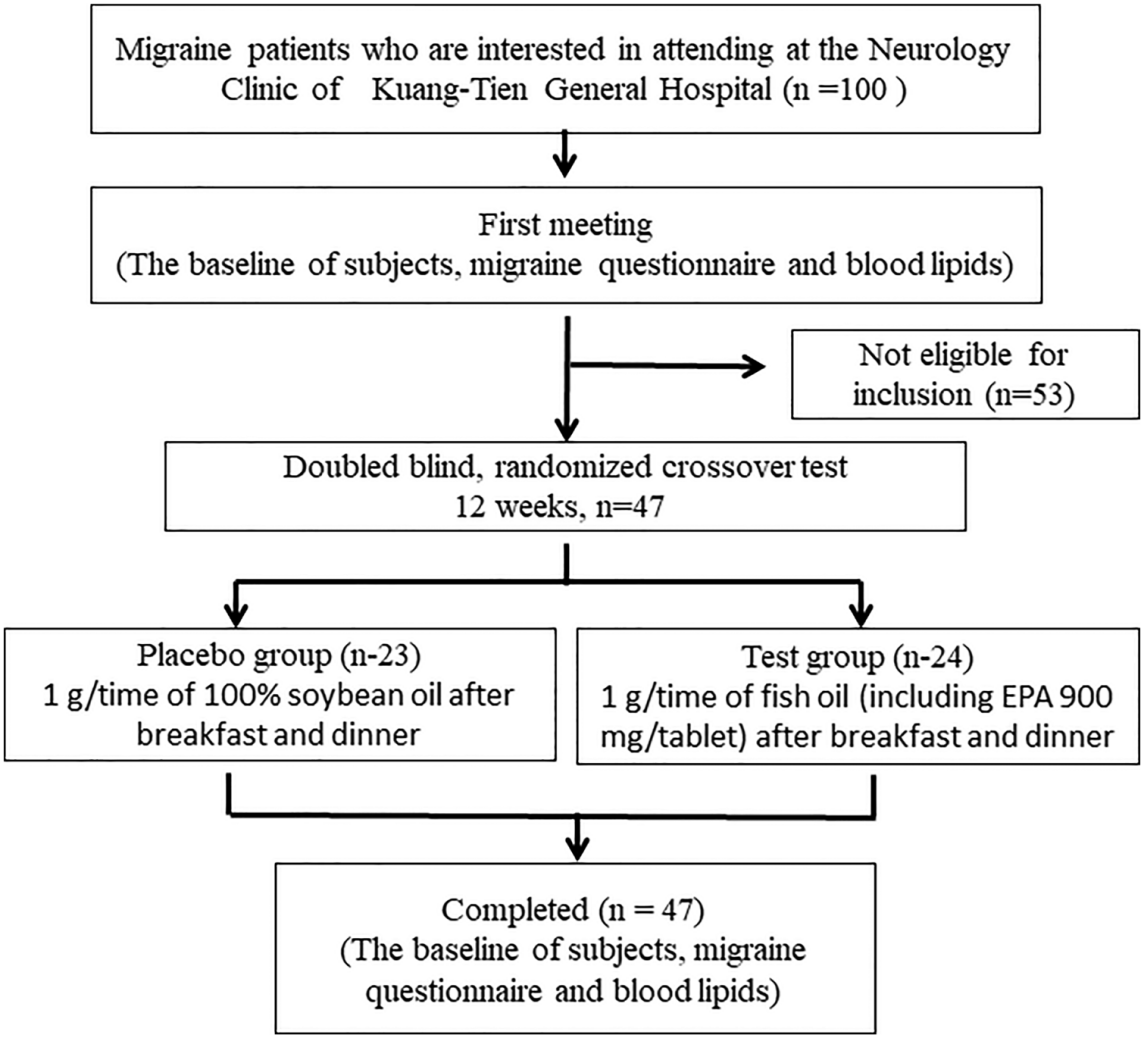
Flow chart of this study.

Figure 1 is the flow chart of this study. This study was a 12‐week randomized and double‐blind clinical trial. The subjects (*n* = 100) expressing interest in involvement were individuals with episodic migraines at the Neurology Clinic of Kuang Tian General Hospital from March 2020 to May 2021. Out of these, 53 did not satisfy the inclusion criteria, whereas 47 fully met the criteria and completed and signed the human trial consent form (KTGH 110844). The inclusion criteria were: (1) aged 20–65 years old, migraine patients were selected based on the third edition of the International Headache Society's diagnostic criteria (Soveyd et al. 2017), which included individuals with both episodic and chronic migraines. The frequency of headaches was recorded as part of the diagnostic assessment; (2) regardless of gender; (3) voluntarily participate in the trial plan after being explained by doctors or planners. The exclusion conditions were: (1) patients with other types of headaches, such as tension‐type headaches, secondary headaches, or any non‐migraine headaches, were excluded from the study; (2) patients with major head trauma in the past; (3) patients with alcoholism within 1 year; (4) patients with poor liver and kidney function (abnormal liver function defined as glutamic oxaloacetic transaminase (GOT)/glutamic pyruvic transaminase (GPT) > 40 IU/L, abnormal renal function defined as creatinine > 1.3 mg/dL, and/or those with other serious diseases such as any infection and inflammation; (5) pregnant women or women who are still breastfeeding; (6) those who cannot cooperate with the progress of the trial; (7) patients with abnormal coagulation function or taking anticoagulant drugs; (8) those who were allergic to fish or fish oil, as well as patients who were regularly taking fish oil supplements; (9) vegetarians; (10) patients who had taken beta blockers, antiepileptic drugs, calcium ion blockers, antidepressants, hormone preparations, or similar medications in the past month were excluded to avoid potential interactions with the study treatment and to ensure that the observed effects could be attributed solely to the intervention. Although this criterion does exclude some individuals, it was necessary to maintain the study's internal validity and accurately assess the effects of the treatment.

### 2.2 Samples and Grouping

In this study, a double‐blind design was employed. Participants were screened and then randomly assigned to either experimental group or placebo group by a designated hospital trial center. To ensure the integrity of the study, neither the participants, the clinical practitioners, nor the assessors were aware of the specific group assignments or whether the groups were receiving a placebo or the experimental intervention. The allocation sequence was managed and safeguarded by the hospital's trial center to prevent any potential biases or breaches in confidentiality. Subjects took two tablets of the test substance daily, one after breakfast and one after dinner, for 12 weeks. The test substance in the experimental group was a 1 g fish oil supplement containing 90% EPA and 10% alpha‐tocopherol equivalent (*α*‐TE) per capsule, and the placebo group was 1 g per capsule containing 100% soybean oil. Samples were purchased from Chen Hua Biotech Business Co., Ltd., Taiwan.

### 2.3 Experimental Measurements

The headache questionnaire assessed the following aspects: (1) headache frequency and duration: Participants provided information on their age at the first headache, age at which headaches worsened, average number of monthly attacks, and the duration of each headache episode; (2) headache intensity: The intensity was evaluated indirectly through questions about the number of pain medications taken per month, which serves as a proxy for headache severity; (3) headache characteristics: This section covered the nature and location of headaches, whether head movement or activity exacerbated the headache, and the presence of associated symptoms such as nausea, vomiting, and sensitivity to light or sound; (4) headache‐related symptoms: The questionnaire included whether migraine attacks were accompanied by nausea, vomiting, loss of appetite, sensitivity to light, sound, or taste; and (5) headache triggers: Participants reported potential triggers including food, drink, taste, and situational factors related to their lifestyle. For experimental analysis, all participants’ 20 mL blood was collected at Weeks 0 and 12. Automatic analysis of fasting blood sugar (FBS), blood lipid parameters (TG, TC, low‐density lipoprotein cholesterol [LDL‐C], high‐density lipoprotein cholesterol [HDL‐C]), liver function parameters (SGOT, SGPT), and renal function parameters (blood urine nitrogen [BUN], creatinine, uric acid) was conducted using a blood biochemical instrument (HITACHI Clinical Analyzer7180, Hitachi Ltd., Tokyo, Japan) (Anon 2018).

The food intake categories of subjects were analyzed by semi‐quantitative food frequency questionnaire (SFFQ) to understand their dietary patterns. The content of the questionnaire includes vegetables, fruits, whole grains, refined starches, dairy products, processed milk, soybeans and processed products, nuts, processed meat, caffeinated foods, artificial sugars, and health foods (fish oil, calcium supplement health food, vitamin B group). The results were quantified and analyzed by the five‐point scale (Likert scale): frequently eaten at least once a day is 5 points, frequently eaten is 4 points, occasionally eaten is 3 points, rarely eaten is 2 points, and do not eat is 1 point, the higher the score, the higher the frequency of eating (Anon 2018).

### 2.4 Statistical Analysis

Data were analyzed using SPSS version 22.0.0 (IBM; Chicago, IL, USA). The level of significance for each statistical procedure is *p* < 0.05. Continuous variables expressed as means and standard deviations were analyzed using the Student's *t*‐test, whereas categorical variables presented as frequencies and percentages were analyzed using the chi‐squared test. The general linear model repeated measure was examined to compare the plasma lipids between the experimental group and the placebo group.

## 3 Results

### 3.1 Subject Characteristics

Table 1 shows that the average age of the 47 subjects in this study was 40.32 ± 9.24 years old, most of them were 31–40 years old, 17.0% were male, and 83.0% were female. The average height was 156.98 ± 24.63 cm; the weight was 59.81 ± 14.87 kg; the body mass index (BMI) was 24.26 ± 6.03 kg/m^2^; there was no significant difference between the experimental group and the placebo group (Table 1). The education level was mostly of university (34.1%); 76.6% were married; most subjects had sun protection (72.3%); daily activities (work). The most of the space light time is more than 20 min under the fluorescent lamp (89.3%); most subjects have no smoking habit (95.7%); no history of depression (93.6%) and poor sleep (53.2%); 1 week in a good mood (68.1%); family history of migraine (51.1%); age of first headache is mostly under 30 years old (74.5%); and age of headache worsening is also under 30 years old.

**TABLE 1:** The basic characteristics of the subjects.

	All subjects *N* = 47	Test group *N* = 24	Placebo group *N* = 23	*p* values
Weight (kg)	59.81 ± 14.87	58.40 ± 17.58	61.29 ± 11.61	0.159
Height (cm)	156.98 ± 24.63	154.71 ± 33.75	159.35 ± 8.09	0.334
Body mass index (kg/m^2^)	24.26 ± 6.03	24.31 ± 7.32	24.24 ± 4.62	—
Age (years)	40.32 ± 9.24	40.42 ± 10.26	40.22 ± 8.28	0.354
Sex man	8 (17.0)	4 (16.7)	4 (17.4)	0.625
Female	39 (83.0)	20 (83.3)	19 (82.6)	—
Married	36 (76.6)	16 (66.7)	20 (87.0)	0.220
Sun protection	34 (72.3)	17 (70.8)	17 (73.9)	0.536
Smoke	2 (4.3)	1 (4.2)	1 (4.3)	0.745
History of depression	3 (6.4)	2 (8.3)	1 (4.3)	0.516
Poor sleep	25 (53.2)	12 (50.0)	13 (56.5)	0.438
Bad mood	15 (31.9)	8 (33.3)	7 (30.4)	0.540
Family history of headache	24 (51.1)	17 (70.8)	7 (30.4)	0.006

*Note*: Values are mean ± SD or *n* (%). Continuous variables were analyzed by Student's *t*‐test. Categorical variables were analyzed by chi‐squared test.

Fish oil and migraine attack triggers, gastrointestinal symptoms, and sensory sensitivities as shown in Table 2. Among the migraine attack trigger factors, situational factors are the most, followed by special smell. After the intervention of fish oil, they were significantly improved (*p* = 0.002, *p* = 0.009), from 123 to 74 and from 33 to 24. For those who developed gastrointestinal symptoms during migraine attacks, there was a significant improvement after fish oil intervention (*p* = 0.015), from 37 to 27. In terms of sensory sensitivity, it decreased from 36 to 31, but there was no significant difference (*p* = 0.239). There was no improvement in the placebo group. The results showed that the experimental group improved gastrointestinal symptoms during migraine attacks better than the placebo group. The above results show that the intervention of fish oil as an adjunctive therapeutic substance is more effective than the placebo group in improving trigger factors, gastrointestinal symptoms, and sensory sensitivity during migraine attacks.

**TABLE 2:** Fish oil and migraine attack triggers, gastrointestinal symptoms, and sensory sensitivities.

	Test group			Placebo group		
	Before *N* = 24	After *N* = 24	*p* values	Before *N* = 23	After *N* = 23	*p* values
Migraine attack triggers						
Food^a^	7	7	1.00	4	4	1.00
Drink^b^	13	7	0.321	7	5	0.184
Special smell^c^	33	24	0.009	16	19	0.501
Situational factors^d^	123	74	0.002	84	77	0.487
Weather change	13	6	0.038	4	7	0.245
Temperature change	10	2	0.009	5	4	0.500
Blowing cold wind	14	7	0.040	10	6	0.177
Sleep too much	9	1	0.005	3	1	0.304
Gastrointestinal symptom sensitivity^e^	37	27	0.015	31	42	0.315
Sensory sensitivity^f^	36	31	0.239	25	23	0.638

*Note*: Values were expressed in numbers of people. The individual calculations were calculated as the individual sums of the factors.

^a, b, c, d, e, f^ Differences between groups were analyzed by chi‐square test, with *p* < 0.05 representing a significant difference.

There was no significant difference in the headache characteristics or locations among migraine subjects before and after the intervention (*p* > 0.05). Specifically, we evaluated 10 types of pain sensations, including pulse‐like pain, persistent pain, neck muscle pain, tingling pain, squeeze pain, tenderness, ice‐chisel‐like pain, tear‐like pain, explosion‐like pain, and throwing pain. Additionally, 12 headache locations were assessed, such as the forehead, temple, top of the head, back of the head, periorbital area, inside or behind the eyeball, around the nose, cheek or jaw, in front of the ear, ear, behind the ear, and back of the neck. Given the nature of the intervention (fish oil), which is believed to have more general effects on inflammatory processes and overall neurological health rather than targeting specific headache characteristics or locations directly, it is not surprising that these aspects did not show significant changes. Our focus was on overall improvements in headache frequency and severity, which were observed, rather than changes in specific headache features. SFFQ analysis of dietary status revealed that subjects with migraine attacks had more frequent intake of refined starches and caffeine foods (4.26 ± 0.98 points, 4.13 ± 1.13 points); less fish oil intake before intervention (1.83 ± 1.37 points). Vegetables, fruits, staple foods, dairy products, soybeans and their processed products, nuts, processed meats, caffeine foods, artificial sugars and health foods (such as vitamin B complex, calcium supplement health food) were having no significant differences in intake frequency and estimated intake (*p* > 0.05).

### 3.2 Episodic Migraine and Plasma Lipids

The plasma lipid levels of the subjects in this study, with the exception of the average total cholesterol in the experimental group (208.75 ± 49.55 mg/dL, which is considered borderline high), were within the normal range. After the fish oil treatment, there was a slight decrease in plasma TG, TC, LDL‐C, and HDL‐C within the experimental group. However, these changes were not statistically significant (*p* > 0.05). It is important to note that these results pertain to the changes observed within the fish oil group before and after treatment. There were no significant differences between the experimental group and the control group in terms of these lipid measures (Table 3).

**TABLE 3:** Effects of fish oil supplementation on blood lipids in patients with migraine.

		Test group		Placebo group				
	Standards (unit)	Before *N* = 24	After *N* = 24	Before *N* = 24	After *N* = 24	*p* values^a^	*p* values^b^	*p* values^c^
TG	30–165 (mg/dL)	98.04 ± 57.62	86.00 ± 54.94	113.35 ± 78.85	106.57 ± 66.62	0.469	0.378	0.954
TC	130–200 (mg/dL)	208.75 ± 49.55	191.46 ± 73.91	198.96 ± 39.42	202.91 ± 33.69	0.534	0.619	0.738
LDL‐C	< 130 (mg/dL)	118.06 ± 54.34	117.14 ± 48.11	114.31 ± 42.46	116.97 ± 39.39	0.487	0.270	0.857
HDL‐C	35–85 (mg/dL)	53.56 ± 12.97	57.34 ± 12.80	52.81 ± 13.17	53.44 ± 13.24	0.561	0.019	0.090
TC/HDL‐C	< 3.5	3.82 ± 1.28	3.81 ± 0.93	3.78 ± 1.09	3.71 ± 0.85	0.072	0.40	0.562

*Note*: Values were expressed in mean ± SD. The authors will get statistical results of within group, between group and also interaction between treatment and time, with *p* < 0.05 representing a significant difference.

Total cholesterol (TC), blood lipid parameters (triglycerides (TG), low‐density lipoprotein cholesterol (LDL‐C), high‐density lipoprotein cholesterol (HDL‐C).

^a^ *p* values for group.

^b^ *p* values for before and after by group.

^c^ *p* values for interaction between treatment and time.

The HDL‐C in both groups were slightly increased; however, there was only significant difference in the experimental group (*p* = 0.019). In the placebo group, only plasma TGs were slightly decreased, and all others were slightly increased, but there were no significant differences. A total cholesterol/high‐density cholesterol ratio (TC/HDL‐C) < 5.0 is considered normal. After 12 weeks of fish oil intervention, the experimental and placebo groups were 3.8 and 3.7, respectively, and there was no significant difference between the two groups before and after the test (Table 3).

### 3.3 Assess Headache Symptoms

Table 4 presents the evaluation results of migraine subjects based on categorical variables such as the frequency and severity of headaches, and painkiller usage. The average number of days per month in which migraine patients took painkillers was fewer than 5 days for 44.7% of the subjects. In the placebo group, there was a statistically significant reduction in the number of times painkillers were taken before and after the test (*p* < 0.05). Regarding the duration of severe headaches without medication, 25.5% of all subjects reported headaches lasting 1–2 days, and this was significantly reduced after the intervention (*p* < 0.05). Similar reductions were observed in both the test group (*p* = 0.001) and the placebo group (*p* = 0.012), though there was no significant difference between the groups at either time point (*p* > 0.05). For the occurrence of severe headaches, 31.9% of subjects reported experiencing more severe headaches at least twice per month. Notably, the experimental group showed significant improvement after the intervention (*p* = 0.002). Over the past 3 months, less than one severe headache was reported by the majority of subjects, with statistically significant improvements across all subjects (*p* = 0.010), the experimental group (*p* = 0.003), and the placebo group (*p* < 0.001). After the intervention, the severity of headaches—rated as 4–6 on a numerical scale—decreased for 15.1% of subjects. Both the experimental and placebo groups showed significant reductions in severity, with all subjects (*p* < 0.001) and the placebo group (*p* < 0.001) demonstrating improvement after the intervention.

**TABLE 4:** Effects of fish oil on headache symptoms.

	Test group			Placebo group		
	Before	After	*p* values	Before	After	*p* values
	*N* = 24	*N* = 24		*N* = 23	*N* = 23	
Average number of headache days per month^b^	16.00	17.33	—	15.33	16.00	—
Non	0 (0.0)	2 (8.3)		0 (0.0)	1 (4.3)	
4–14 days	24 (100.0)	22 (91.7)		23 (100.0)	22 (65.7)	
Average number of days a month with pain medication^b^	31.20	33.60	0.001^a^	28.00	28.00	0.291
Non	9 (37.5)	12 (50.0)		8 (34.8)	13 (56.5)	
≤ 5 days	13 (54.2)	12 (50.0)		8 (34.8)	4 (17.4)	
6–10 days	1 (4.2)	0 (0.0)		7 (30.4)	0 (0.0)	
≥ 11 days	1 (4.2)	0 (0.0)		0 (0.0)	6 (26.1)	
Without medication, usually the duration of each more severe headache^b^	18.40	28.00	0.001^a^	18.80	23.20	0.012^a^
< 30 min	3 (12.5)	13 (54.2)		4 (17.4)	9 (39.1)	
30–240 min	4 (16.7)	2 (8.3)		3 (13.0)	2 (8.7)	
4–24 h	5 (20.8)	3 (12.5)		6 (26.1)	6 (26.1)	
≥ 1 day	12 (50.0)	6 (25.0)		10 (43.5)	6 (26.1)	
Average monthly occurrences of this more severe headache in the past^b^	20.80	29.60	0.002^a^	21.60	23.60	0.347
≤ 1	4 (16.7)	12 (50.0)		6 (26.1)	6 (26.1)	
1	3 (12.5)	4 (16.7)		4 (17.4)	7 (30.4)	
2	10 (41.7)	6 (29.1)		5 (21.7)	4 (17.4)	
≥ 3	7 (29.2)	2 (8.3)		8 (34.8)	6 (26.1)	
How severe do you think the more severe headaches have occurred in the past three months^b^	22.8	24.4	< 0.001^a^	25.60	28.00	0.003^a^
0	0 (0.0)	0 (0.0)		0 (0.0)	1 (4.2)	
1–3	15 (65.2)	17 (73.9)		17 (70.8)	21 (87.5)	
4–6	4 (17.4)	4 (17.4)		6 (25.0)	1 (4.2)	
7–10	4 (17.4)	2 (8.7)		1 (4.2)	1 (4.2)	
Your average headache over the past three months and its severity^b^	18.4	23.6	< 0.001^a^	18	17.6	0.121
0	1 (4.2)	2 (8.3)		1 (4.3)	1 (4.3)	
1–3	4 (16.7)	11 (45.8)		3 (13.0)	3 (13.0)	
4–6	11 (45.8)	7 (29.2)		13 (56.5)	12 (52.2)	
7–10	8 (33.3)	4 (16.7)		6 (26.1)	7 (30.4)	

*Note*: Values are expressed in number of people (%).

^a, b^ Differences between groups were analyzed by chi‐square test, when *p* < 0.05 representing a significant difference.

## 4 Discussion

### 4.1 Migraine Triggers and Fish Oil Intervention

Migraine attacks not only impact the individuals experiencing them but also extend their influence on family and friends, often leading to social disruption, anxiety, and fear. Many patients opt for treatment when migraines interfere with their daily life and work (Steiner et al. 2014). As shown in Table 1, the data reveal that 17.0% of men and 83.0% of women proactively sought treatment at the hospital's outpatient clinic, indicating a higher proportion among women. Epidemiological studies emphasize significant gender disparities in migraine incidence (Singh and Singh 1947; Waters and O'Connor 1975), particularly among women. Migraine attacks in women typically commence after the first menstrual period, with heightened occurrence before and after menstruation (Sacco et al. 2012). Clinical findings indicate that women are three times more likely than men to experience migraines during the menstrual period, known as premenstrual syndrome (PMS) (MacGregor 2004). Menstrual migraines typically manifest 2 days before the menstrual period and persist until the third day after. During this phase, decreased estrogen secretion from the ovaries affects the brain's nerve conduction system, leading to diminished serotonin secretion. This decrease prompts the trigeminal nerve to release a protein that lowers its pain perception threshold, resulting in more tolerable pain. Concurrently, cerebral blood vessels dilate, giving rise to migraine headaches (Moy and Gupta 2022). Menstruation also involves the endometrium secreting prostaglandins, promoting uterine contractions, and menstrual blood discharge. However, prostaglandins also induce cerebral vasoconstriction, contributing to headaches during this period (Barcikowska et al. 2020).

Exposure to light plays a crucial role in triggering migraine headaches. Typically, in countries with higher latitudes and limited access to sunlight, there is a relatively high prevalence of migraines, with a greater frequency observed in autumn and winter compared to spring and summer (Prakash et al. 2010). The participants in this study also experienced a generally shorter duration of exposure to light. Following a 12‐week intervention with fish oil, all participants showed improvement in migraine‐triggering factors such as weather changes, temperature fluctuations, cold winds, and excessive sleepiness (refer to Table 1). Notably, the category of weather changes exhibited significant differences (*p* < 0.05), with all subjects in the experimental group demonstrating significant improvement (*p* < 0.05). However, there was no significant difference observed in the placebo group (*p* > 0.05).

### 4.2 Sleep, Sensory Perception, and Lifestyle Factors

Many women experiencing migraines before and after their menstrual period exhibit characteristics of tension‐type individuals, displaying signs of mild depression, frequent late nights, insufficient sleep, and heightened stress levels. Particularly during migraine attacks, the subjects often endure poor sleep quality, exacerbating their headaches (Vgontzas and Pavlović 2018). In this study, a higher proportion of participants, specifically 53.2%, reported not typically enjoying restful sleep. The presence of migraines was found to be associated with a family history of genetics, as indicated in Table 1. Genetic factors play a role in predisposing individuals to migraines, with common hereditary types including migraine without aura and migraine with aura (Sutherland, Albury, and Griffiths 2019).

From a clinical standpoint regarding migraine triggers, sensory perceptions become heightened during or preceding a migraine attack, with a particular emphasis on the sense of smell, including stimuli such as cigarettes, perfume, and bleach. Migraine patients are prone to headaches triggered by specific odors, and notable differences exist between boys and girls in response to perfume and cigarette scents (Robbins 1994). In this study, the three most prevalent taste‐induced migraine triggers were 36.2% in confined spaces, 25.5% related to perfume, and 23.4% associated with cigarette or cigar odors.

Migraine headaches can be triggered by various factors, including the consumption of caffeinated foods, processed foods, substance abuse, changes in sleep habits, weather fluctuations, alterations in female hormones (related to menstruation and menopause), obesity, stress, and neck pain (Bigal, Liberman, and Lipton 2006; Imai et al. 2023). Among patients, the most prevalent triggers for migraines were related to diet (84.5%), sleep (75.5%), environment (68.5%), stress (65.0%), hormones (43.5%), and exercise (15.5%) (Fukui et al. 2008). In a study by Robbins scholars, stress (62.0%) and weather changes (43.0%) were identified as the top two factors inducing migraines among 494 patients they investigated (Robbins 1994). According to Table 2, the three primary triggers for migraines in the participants were insufficient sleep or insomnia (74.5%), stress (51.1%), and physical fatigue or exhaustion (44.7%). Issues such as sleep deprivation, stress, and physical fatigue all demonstrated improvement after the 12‐week trial, with the experimental group exhibiting better results than the placebo group, aligning with findings from Robbins’ research.

Dietary patterns that include alcohol and caffeine are commonly linked to an increased frequency of migraine attacks, particularly those involving caffeinated chocolate (Park et al. 2016). Epidemiological reports indicate a correlation between chocolate consumption and migraine attacks in individuals with chronic migraine (Martin and Vij 2016). Alcohol, chocolate, and coffee are frequently associated with migraine episodes (Robbins 1994; Chabriat et al. 1999). A low‐fat diet and a food elimination diet have been shown to reduce the frequency of migraine attacks in sufferers (Hindiyeh et al. 2020). However, even after the intervention with fish oil in this study, 20.8% of migraine patients in the experimental group still experienced headaches after consuming alcoholic beverages, and 8.3% of those who consumed caffeinated beverages had migraine attacks. This suggests that individuals undergoing episodic migraine treatment should avoid the consumption of alcoholic or caffeinated beverages.

Moreover, foods that increase serotonin levels, such as deep‐sea fish rich in fish oil, chicken, beef, nuts, bananas, spinach, milk, and carbohydrates (e.g., whole‐wheat bread, soda crackers), are associated with pleasure (Wurtman and Wurtman 1995). In the current study, individuals with episodic migraines reported a lower daily intake frequency of vegetables, non‐citrus fruits, nuts and seeds, and fresh milk. Notably, those with more severe monthly headache days and greater average headache severity exhibited lower intake frequencies of these foods (*p* < 0.05). Additionally, a higher frequency of consuming processed dairy products was correlated with more severe headache intensity (*p* < 0.05). Following a 12‐week intervention with fish oil, an improvement in the severity of more severe headaches was observed with an increased frequency of fish oil food intake. Consequently, the daily supplementation of fish oil–based health food may have a preventive and ameliorative effect on migraine attacks.

### 4.3 Cardiovascular Implications and Fish Oil Benefits

The association between migraine and cardiovascular disease has been extensively discussed (Kurth et al. 2016; Schürks et al. 2009), and studies by Assarzadegan et al. have indicated higher TC values in migraine patients, correlating with increased plasma TC values (Assarzadegan et al. 2019; Scher et al. 2005). However, effective migraine treatment has been shown to reduce the elevated plasma TC (Tana et al. 2015). Plasma TC and TGs exhibit a significant positive association with migraine attack frequency in women (Janoska, Chorążka, and Domitrz 2015). Considering these findings, elevated plasma TC and TG may be crucial factors contributing to the severity of migraines in women. Consequently, high plasma TC, LDL‐C, and TG levels may elevate the risk of cardiovascular disease in migraine patients, while an increase in HDL‐C can lower this risk.

Moreover, the total cholesterol/high‐density cholesterol ratio (TC/HDL) serves as a risk indicator for predicting cardiovascular disease and metabolic syndrome. A normal value is <5.0, and when the ratio is > 3.5 in men or TC in women, the risk of cardiovascular disease significantly rises. A cholesterol/HDL‐C ratio > 2.5 indicates a substantially increased risk of cardiovascular disease. If the TC/HDL‐C ratio is between 5.0 and 6.0, there is a risk of coronary heart disease, whereas a TC/HDL‐C ratio > 6 suggests a high risk of coronary heart disease (Kromhout et al. 2012). In this study, the subjects’ TC/HDL‐C index ranged around 3.7–3.8, still within the normal range but deserving attention.

According to a 2002 American College of Cardiology (ACC) study, which presented findings from two large randomized controlled trials (RCT), supplementation of EPA and DHA can significantly reduce the incidence of fatal heart disease (Burr et al. 1989). As a result, omega‐3 PUFA may decrease the risk of thrombosis by inhibiting platelet aggregation, enhancing vascular endothelial function, reducing TG levels, and lowering blood pressure (Kromhout et al. 2012).

### 4.4 Impact on Lipid Profiles

In this study, the TC levels in migraine subjects exceeded the standard value, and after 12 weeks of supplementation, the TC in the experimental group was lower than that in the placebo group. Additionally, LDL‐C values were lower in the experimental group compared to the placebo group after 12 weeks. LDL‐C plays a role in transporting cholesterol from the liver to surrounding tissues, where it can accumulate in the lining of blood vessels, leading to coronary arteriosclerosis and heart disease. Elevated LDL‐C levels pose a risk for conditions such as hyperlipidemia, arteriosclerosis, and myocardial infarction. The research findings of Hartweg et al. (2017) demonstrated that supplementing with 3.5 g/day of fish oil in patients with Type 2 diabetes significantly reduced TC, although HDL‐C, FBS, and TC did not show significant changes. In the present study, after 12 weeks of fish oil intervention, although TC, LDL‐C, TG, and FBS did not exhibit significant differences from Week 0, they all demonstrated significant improvement.

Fish oil provides cardiovascular health benefits primarily because EPA and DHA exhibit anti‐inflammatory properties and improve vasoconstriction and dilation (Cottin, Sanders, and Hall 2011). These effects contribute to the overall well‐being of the cardiovascular system. The US Food and Drug Administration (FDA) recommends a daily fish oil supplementation of 4 g as a reference therapy for patients with hypertriglyceridemia (Zibaeenezhad et al. 2017). In the current study, the fish oil supplementation dosage was 2 g/day, which may have had a reduced effect compared to the recommended dosage. Therefore, if migraine patients extend the duration of fish oil supplementation, it is likely to have a more significant impact on their health.

Prior research has predominantly focused on low‐concentration mixed fish oil, with numerous studies indicating the positive impact of fish oil on inflammatory diseases, including a reduction in the frequency of headaches per month in migraine patients (Cardia et al. 2020; Calder 2010; Soveyd et al. 2017). However, there has been limited exploration of high‐concentration fish oil in the literature. Hence, this study was undertaken to investigate the therapeutic effects of fish oil on clinical symptoms in patients with migraine, specifically comparing the improvements before and after intervention in adults.

The study results revealed that individuals with episodic migraines who received a daily dose of 2 g of fish oil experienced a significant decrease in various migraine‐related parameters after 12 weeks. These included the average number of headaches per month, the average number of days requiring pain medication per month, the average number of severe headaches per month, and the average severity of headaches over the last 3 months. Notably, there was a significant reduction in the severity of headaches in the last 3 months, as well as in the severity of more intense headaches (*p* < 0.05).

### 4.5 Fish Oil's Therapeutic Potential for Migraines

Research has demonstrated that a daily supplementation of 1 g of fish oil for 2 months can effectively reduce the frequency and duration of migraine headaches by 74%. (Soveyd et al. 2017). In a study by Tajmirriahi et al., the combination of sodium valproate and fish oil intervention was compared to sodium valproate alone. The results indicated a significant reduction in headache duration, severity, and frequency in the group that received fish oil intervention alongside sodium valproate. This suggests that the combination of fish oil with sodium valproate may be more effective in helping patients suppress migraine attacks (Tajmirriahi et al. 2012).

In the present study, the experimental group underwent a 12‐week intervention with fish oil supplementation, resulting in a significant decrease in the severity of more intense headaches (*p* < 0.01) and a reduction in the frequency of headaches. These findings align with previous research, supporting the potential effectiveness of fish oil supplementation in mitigating migraine symptoms.

The findings of this study can provide valuable insights for migraine patients and their families, offering a better understanding of the positive impact of fish oil intervention on migraine symptoms. This information can be particularly helpful for individuals experiencing migraine attacks and may contribute to a greater understanding of their specific migraine triggers. Participants in this study benefited from regular interactions with physicians, ensuring comprehensive medical care. The study revealed that fish oil intervention resulted in better headache symptoms and improved blood lipid levels compared to the placebo group. This suggests that fish oil can effectively assist migraine patients in reducing or ameliorating migraine‐related symptoms, aligning with the study's intended goals.

To further enhance the quality of life for migraine patients, it is recommended to incorporate fish oil supplementation alongside medical treatment and medication. This approach can not only help alleviate and improve headache‐related symptoms but also potentially reduce healthcare expenses, contributing to the overall maintenance of a higher quality of life for migraine patients.

The primary limitation of this study is the small sample size, as patient expectations for rapid improvement in drug treatment led to fewer individuals willing to participate in the case acceptance. Additionally, the patients in this study generally did not utilize drug therapy, resulting in a slower‐than‐expected response effect. To address these limitations and enhance the robustness of future studies, it is recommended to consider interventions involving drug therapy. This approach may facilitate a larger and more diverse participant pool and potentially yield faster and more pronounced improvements, providing a more comprehensive understanding of the effects of fish oil supplementation on migraine patients.

## 5 Conclusion

The above results found that (1) fish oil can effectively reduce the frequency, duration, and pain of migraine attacks; (2) fish oil can increase plasma HDL‐C and reduce the effect of plasma TC, LDL‐C, and TG in patients with migraine attacks; (3) more intake of vegetables, fish oils other than citrus fruits and health foods can reduce migraine attack frequency, duration, pain intensity, and days on pain medication. Therefore, fish oil supplementation has been shown to reduce the average number of headache days per month, the number of days on pain medication, the average number of more severe headaches, the more severe headache severity, and the average headache severity.

## Author Contributions

**En‐Ling Yeh**: formal analysis. **Chun‐Pai Yang**: data curation. **Shu‐Wen Lin**: formal analysis. **Hsueh‐Fang Wang**: conceptualization, investigation, project administration.

## Ethics Statement

All procedures performed in studies involving human participants were in accordance with the ethical standards of the institutional and/or national research committee and with the 1964 Helsinki Declaration and its later amendments or comparable ethical standards.

## Conflicts of Interest

The authors declare no conflicts of interest.

### Peer Review

The peer review history for this article is available at https://publons.com/publon/10.1002/brb3.70149.

## Data Availability

The data that support the findings of this study are available from the corresponding author upon reasonable request.
